# Comparative molecular analyses of invasive fall armyworm in Togo reveal strong similarities to populations from the eastern United States and the Greater Antilles

**DOI:** 10.1371/journal.pone.0181982

**Published:** 2017-07-24

**Authors:** Rodney N. Nagoshi, Djima Koffi, Komi Agboka, Kodjo Agbeko Tounou, Rahul Banerjee, Juan Luis Jurat-Fuentes, Robert L. Meagher

**Affiliations:** 1 Center for Medical, Agricultural and Veterinary Entomology, United States Department of Agriculture-Agricultural Research Service, Gainesville, Florida, United States of America; 2 Africa Regional Postgraduate Programme in Insect Science, University of Ghana, Accra, Ghana; 3 Ecole Supérieure d'Agronomie, Université de Lomé, Lomé, Togo; 4 Department of Entomology and Plant Pathology, University of Tennessee, Knoxville, Tennessee, United States of America; Universidade Federal de Vicosa, BRAZIL

## Abstract

The fall armyworm (*Spodoptera frugiperda*, J.E. Smith) is a noctuid moth that is a major and ubiquitous agricultural pest in the Western Hemisphere. Infestations have recently been identified in several locations in Africa, indicating its establishment in the Eastern Hemisphere where it poses an immediate and significant economic threat. Genetic methods were used to characterize noctuid specimens infesting multiple cornfields in the African nation of Togo that were tentatively identified as fall armyworm by morphological criteria. Species identification was confirmed by DNA barcoding and the specimens were found to be primarily of the subgroup that preferentially infests corn and sorghum in the Western Hemisphere. The mitochondrial haplotype configuration was most similar to that found in the Caribbean region and the eastern coast of the United States, identifying these populations as the likely originating source of the Togo infestations. A genetic marker linked with resistance to the Cry1Fa toxin from *Bacillus thuringiensis* (*Bt*) expressed in transgenic corn and common in Puerto Rico fall armyworm populations was not found in the Togo collections. These observations demonstrate the usefulness of genetic surveys to characterize fall armyworm populations from Africa.

## Introduction

The fall armyworm, *Spodoptera frugiperda* (J.E. Smith), is the primary pest of corn production in South America and in portions of the southeastern United States [1]. Although it is unable to survive freezing winters, fall armyworm infestations extend as far north as Canada, the result of annual long-distance migrations from overwintering areas in southern United States and Mexico [2–4]. In 2016, severe outbreaks of fall armyworm were reported in several western and central African countries, representing the first indication of the species establishing itself in the Eastern Hemisphere [5]. The voracious feeding and long-distance flight behaviors exhibited by fall armyworm indicate a significant threat to African agriculture with the potential for rapid dispersion throughout the hemisphere.

Fall armyworm consists of two subpopulations that differ in host plant distribution and certain physiological features but are morphologically indistinguishable [6–9]. Larvae collected from rice and corn were found to differ with respect to molecular markers, and were designated as "rice-strain" or "corn-strain". Subsequent studies found that the rice-strain is most consistently found in millet and grass species associated with pasture habitats while the corn-strain prefers corn and sorghum [10–12]. Strain differences have also been reported in female pheromone composition, mating behavior, and physiology, though there appears to be substantial variability or plasticity in these phenotypes [8, 13–16].

At this time, genetic polymorphisms are the most reliable method of identifying strains. Mitochondrial haplotypes are most commonly used, with those defined by polymorphisms in the *Cytochrome oxidase subunit I* gene (*CO1*) the best characterized ([17–19]). Strain-specific markers in the nuclear genome appear to be rare and are currently limited to a small number of polymorphic loci located on the *Z-*chromosome ([20–22]). An example are single-nucleotide polymorphisms (SNPs) in the gene encoding for *Triosephosphate isomerase* (*Tpi*) that generally show a stronger correlation with host plants than the *CO1* strain haplotypes, perhaps indicating more accurate identification of strains than the mitochondrial markers [21, 23, 24]. These observations illustrate that the strain-specific associations observed with existing markers are not absolute. Discordances between host plant to molecular markers and between different markers have been observed in multiple locations and can be substantial [11, 22, 25, 26]. The reasons for this variability is unknown, but contributing factors could include incomplete fixation of the markers to the two strains, variability in strain behaviors, or hybridization between strains. So while there is good evidence for the existence of the two strains throughout the Western Hemisphere, we can currently only approximate the strain identity of any given specimen.

The corn-strain population based on the *CO1* markers can be subdivided into two geographically distinct subgroups on the basis of differences in the frequency of certain haplotypes [27]. Corn-strain fall armyworm that overwinter in southern Texas (TX) share the same set of haplotypes in the mitochondrial *Cytochrome oxidase subunit I* (*CO1*) gene as those found in overwintering populations in Florida (FL), but differ in their relative frequency. This haplotype distribution difference is sufficiently reproducible to allow mapping of the migratory populations that emanate from the two overwintering locations [28]. Overall, the TX haplotype profile is found throughout most of the Western Hemisphere, with the FL profile limited to the eastern coast of the U.S. from Maryland to Florida and extending southward to Puerto Rico and the Lesser Antilles [29–31]. Analogous haplotype differences for the rice-strain have not yet been found, so it is unclear whether this group shows a similar geographical distribution.

Another geographically defined marker has recently been characterized. In 2006, field-evolved resistance to transgenic corn expressing the *Bacillus thuringiensis* (*Bt*) toxin Cry1Fa was reported in fall armyworm populations from Puerto Rico [32]. Subsequently, resistance to Cry1Fa corn was also found in the southeastern United States and Brazil, though the relationship with the Puerto Rico trait has not been established [33, 34]. Recently, the Puerto Rico resistance allele (*SfABCC2mut*) was identified (Banerjee et al., *in review*), providing a means to detect this resistance trait by genetic methods.

In this paper we analyze specimens from several agricultural regions in the African nation of Togo collected in the latter half of 2016. Genetic analyses confirmed the fall armyworm identification of the specimens, estimated host strain identity, and tested for the presence of the Puerto Rico *Bt*-resistance allele. The haplotype and marker data were used to extrapolate the most likely Western Hemisphere source locations. The ramifications of these results on the pest potential of the Togo fall armyworm population are discussed.

## Materials and methods

### Specimen collections and DNA preparation

**Fig 1:**
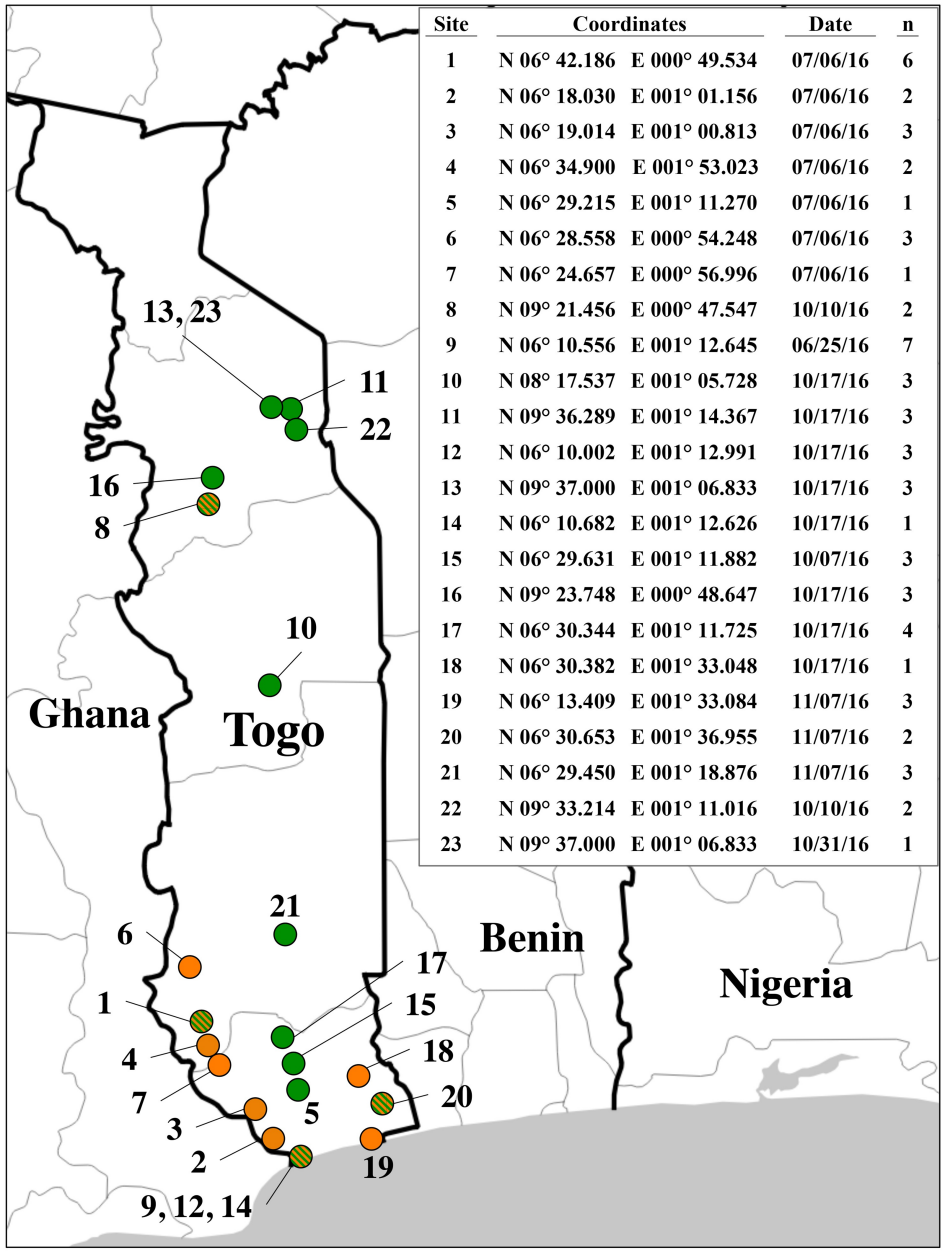
Map of Togo showing location of fall armyworm collection sites. The embedded table provides coordinate information, collection date, and the number of specimens (n) tested from each site. Color of circles indicate strain found at each site based on the *CO1* barcode analysis; Green, only *CO1*-CS; orange, only *CO1*-RS; orange and green, both *CO1*-CS and *CO1*-RS found.

Specimens were obtained as larvae from corn (maize) plants at various locations in Togo, Africa from July to November 2016 (Fig 1). Specimens were stored either air-dried or in ethanol at room temperature. A portion of each specimen was excised and homogenized in 1.5 ml of phosphate buffered saline (PBS, 20 mM sodium phosphate, 150 mM NaCl, pH 8.0) using a tissue homogenizer (PRO Scientific Inc., Oxford, CT, USA) and the homogenate transferred to a 2-ml microcentrifuge tube. The unused portion was stored in ethanol at -20°C. The homogenized tissue was pelleted by centrifugation at 6000 x *g* for 5 min. at room temperature and the pellet resuspended in 800 μl of Genomic Lysis buffer (Zymo Research, Orange, CA, USA) and incubated at 55°C for 5–30 min. Debris was removed by centrifugation at 10,000 rpm for 3 min. The supernatant was transferred to a Zymo-Spin III column (Zymo Research, Orange, CA, USA) and processed according to manufacturer’s instructions. The DNA preparation was increased to a final volume of 100 μl with distilled water.

### Characterization of the *CO1* and *Tpi* gene segments

**Fig 2:**
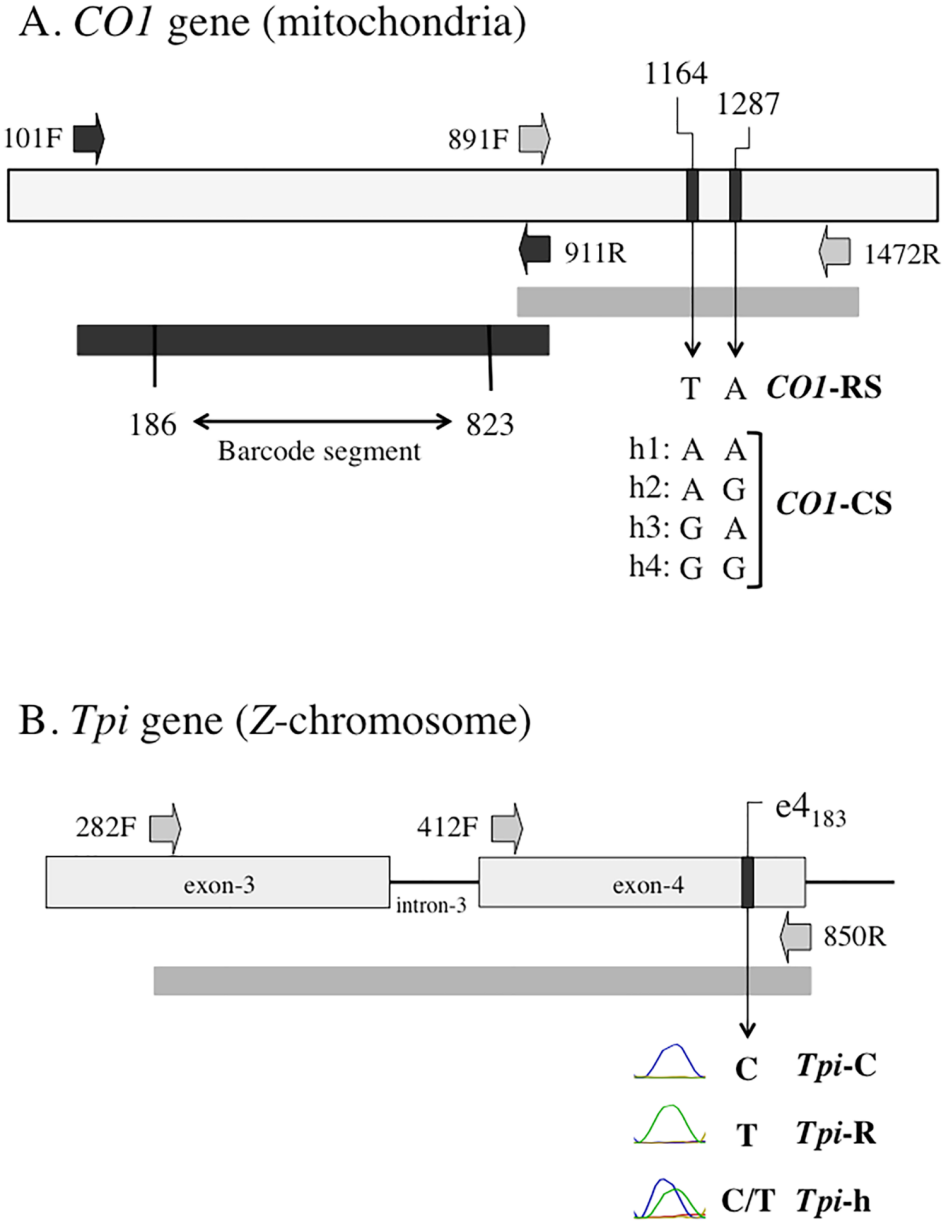
Diagrams of the segments from the *COI* and *Tpi* genes used for the genetic analysis. A: *CO1* gene segment with dark block arrows identifying PCR primers used to amplify the fragment (dark bar) that contains the barcode region (from nucleotide 186 to 823). Grey block arrows identify primers used to amplify the fragment (light bar) with the *CO1*_1164_ and *CO1*_1287_ polymorphic sites. *CO1*-RS is defined as a T at *CO1*_1164_ together with an A at *CO1*_1287_. There are four corn-strain (*CO1*-CS) haplotypes (h1-h4) with and A or G at both *CO1*_1164_ and *CO1*_1287_. B: Portion of the fall armyworm *Tpi* gene with block arrows indicating PCR primers. The e4_183_ site is polymorphic for a C or T. PCR amplification was done using primers 282F and 850R. DNA sequencing was performed using primer 412F, which initiates in the same exon as e4_183._ Representative DNA chromatograph patterns are shown to illustrate how *Tpi*-C, *Tpi*-R, and *Tpi*-h are defined.

The *CO1* markers are from the mitochondrial genome and so are maternally inherited. Two adjacent segments of *CO1* were analyzed by DNA sequencing (Fig 2A). The segment amplified by the *CO1* primers 101F and 911R was used to identify species and fall armyworm host strain (*CO1* corn-strain is designate *CO1*-CS, *CO1* rice-strain as *CO1*-RS). The DNA sequences of the fall armyworm host strains and other *Spodoptera* species were previously described and available in GenBank [35]. DNA alignments and consensus building were performed using MUSCLE (multiple sequence comparison by log-expectation), a public domain multiple alignment software incorporated into the Geneious Pro 10.1.2 program (Biomatters, New Zealand, http://www.geneious.com, [36]). Phylogenetic trees were graphically displayed in a neighbor-joining (NJ) tree analysis also included in the Geneious Pro 10.1.2 program [37].

The adjacent segment amplified by *CO1* primers 891F and 1472R was used to confirm host strain identity and determine the region-specific haplotype metric [38] (Fig 2A). DNA sequence analysis of the SNPs at sites *CO1*_*1164*_ *and CO1*_*1287*_ identifies one rice-strain (*CO1*_*1164*_ = T, *CO1*_*1287*_ = A) and four corn-strain haplotypes (h1-h4) defined by polymorphisms at two loci that together encompass the corn-strain group (Fig 2A). Haplotypes h1 and h3 are generally infrequent, while h2 and h4 frequencies vary by region, with h2 predominant in Texas and h4 the majority in Florida. The haplotype profiles of the collections were compared by the metric (h4 –h2)/(h4 + h2) that varies from a minimum of -1 (all h2) to a maximum of +1 (all h4). The haplotype profile is categorized as the FL-type when the ratio is greater than or equal to 0.1, the TX-type when the ratio is less than or equal to -0.3, and a "mixed" profile called FAW[M] with intermediate values, -0.3 < ratio < 0.1 that is suggested to arise from the mixing of the TX-type and FL-type groups (described in [38]).

Polymorphisms in the *Tpi* gene can also be used to identify host strain identity with results that compare favorably with the *CO1* marker [21, 23]. The e4_183_ polymorphic site is on exon4, 183 bp downstream of the 5' splice site. Previously denoted as C370, e4_183_ is one of 10 SNPs in the *Tpi* segment spanning exons 3 and 4 (including the intervening intron) that we showed had a strain bias [23]. We further demonstrated that the e4_183_ SNP alone gave strain identification results that were not significantly different than that based on all 10 strain-biased SNPs [23]. The e4_183_ SNP varies between a C or T, indicating either the corn-strain (*Tpi*-C) or rice-strain (*Tpi*-R), respectively.

Because of its location on the Z chromosome, the direct sequencing of the PCR product amplified from male genomic DNA will represent an overlap of two *Tpi* genes. This is particularly problematic if the region being sequenced is heterozygous for an insertion or deletion, which causes a misalignment of the sequencing frame and ambiguous sequence data. To minimize this problem, the PCR product from primers 282F and 850R was sequenced with primer 412F, which lies in the same e4 exon as e4_183_ (Fig 2B). All heterozygous polymorphisms observed within the e4 exon were SNPs, which at e4_183_ was indicated by an overlapping C and T DNA sequence chromatograph and denoted as *Tpi*-h (Fig 2B).

PCR amplification for all segments was performed in a 30-μl reaction mix containing 3 μl 10X manufacturer’s reaction buffer, 1 μl 10mM dNTP, 0.5 μl 20-μM primer mix, 1 μl DNA template (between 0.05–0.5 μg), 0.5 unit Taq DNA polymerase (New England Biolabs, Beverly, MA). The thermocycling program was 94°C (1 min), followed by 33 cycles of 92°C (30 s), 56°C (45 s), 72°C (45 s), and a final segment of 72°C for 3 min. Typically 96 PCR amplifications were performed at the same time using either 0.2-ml tube strips or 96 well microtiter plates. All primers were obtained from Integrated DNA Technologies (Coralville, IA). Amplification of the *CO1* barcode region was performed using primers *101F*, 5’- TTCGAGCTGAATTAGGGACTC -3’ and *COI-911R* (5’- GATGTAAAATATGCTCGTGT -3’ to produce an 811 bp fragment (Fig 1). Amplification of the *CO1* segment used to determine the haplotype metric used the primer pair *891F* (5’-TACACGAGCATATTTTACATC-3’) and *1472R* (5’-GCTGGTGGTAAATTTTGATATC-3’) to produce a 603-bp fragment. Amplification of the *Tpi* gene segment used the primers *282F* (5’-GGTGAAATCTCCCCTGCTATG -3’) and *850R* (5’- AATTTTATTACCTGCTGTGG -3’) that spans a variable length intron to produce a fragment with an approximate length of 500 bp.

For fragment isolations, 6 μl of 6X gel loading buffer was added to each amplification reaction and the entire sample run on a 1.8% agarose horizontal gel containing GelRed (Biotium, Hayward, CA) in 0.5X Tris-borate buffer (TBE, 45 mM Tris base, 45 mM boric acid, 1 mM EDTA pH 8.0). Fragments were visualized on a long-wave UV light box and manually cut out from the gel. Fragment isolation was performed using Zymo-Spin I columns (Zymo Research, Orange, CA) according to manufacturer’s instructions. The University of Florida Interdisciplinary Center for Biotechnology (Gainesville, FL) and Genewiz (South Plainfield, NJ) performed the DNA sequencing.

### Genotyping for resistance to *Bt* corn

The genotyping test detecting the *SfABCC2mut* allele linked to resistance against Cry1Fa corn in fall armyworm from Puerto Rico has been recently described (Banerjee et al., *in review*). Genomic DNA isolated as described above (5–7.5 ng) was used as template for 10 μl Taqman^®^ custom SNP Genotyping (Invitrogen, Carlsbad, CA) reactions in wells of a Micro Amp Fast optical 96 well reaction plate (Applied Biosystems, Foster City, CA). Reactions included a VIC-labeled probe specific to the mutant (resistant) allele (5’-AAGCACATCGCCCACTT-3’), a FAM-labeled probe specific to the wild type allele (5’-CCAAGCACATCCCACTT-3’), and forward (5’-TGGAGGCCGAAGAGAGACA-3’) and reverse (5’-AGGAGTTGACTGACTTCATGTACCT-3’) primers. Controls included genomic DNA from homozygous susceptible (wild type), homozygous resistant (*SfABCC2mut*), and hybrid (wild type/*SfABCC2mut*) individuals, as described in Banerjee et al (*in review*). The plate was run in a Quant studio 6 Real Time PCR instrument (Applied Biosystems, Foster City, CA) using the following conditions: pre read stage at 60°C for 30 seconds, hold stage at 95°C for 10 minutes, PCR stage at 95°C for 15 seconds and 60°C for 1 minute for 40 cycles, post read stage at 60°C for 30 seconds. The fluorescence in each well was measured in the post read stage of the PCR. The allelic discrimination plot generated from the post amplification intensity of the fluorescent probes was used to determine the genotype of each sample.

## Results

### Togo specimens are predominantly corn-strain fall armyworm

**Fig 3:**
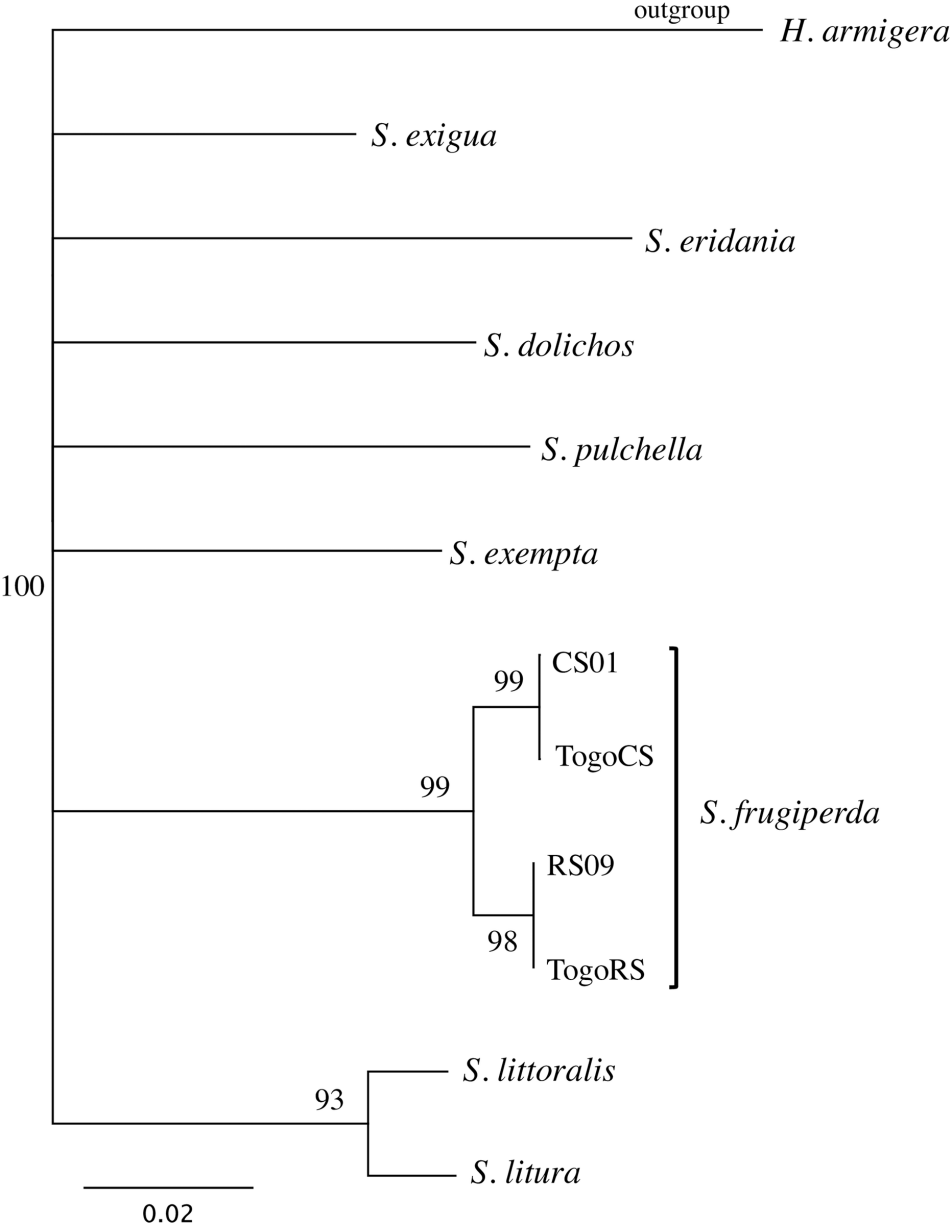
Strict consensus phylogenetic tree derived from neighbor- joining analysis comparing the two Togo barcode sequences (Togo CS, Togo RS) with those from fall armyworm host strains and related *Spodoptera* species [35]. The sequences from the *Spodoptera* species are the consensus of the following variants found in Genbank, *S*. *dolichos* (HM756086-9), *S*. *eridania* (HM756081-5), *S*. *exempta* (HQ177331-6, DQ092371-6), *S*. *exigua* (HM756077-80), *S*. *litura* (HM6090-3), *S*. *littoralis* (HM756074), *S*. *pulchella* (756075–6). The two distinct Togo sequences were identical to the fall armyworm corn strain haplotype CSO1 (HM136586) and rice strain haplotype RS01 (HM136601). The phylogenetic analysis was based on a 402-bp segment of the *CO1* gene common to all sequences with the equivalent *CO1* segment from *Helicoverpa armigera* (KM275101) used as the outgroup. The tree is based on Kimura-2-Parameter distances. Numbers at branch points indicate 2000X bootstrap values. Scale bar represents substitutions per site.

The *CO1* region frequently used for DNA barcoding was previously shown to distinguish between closely related *Spodoptera* species, including differentiating between the two fall armyworm host strains [35]. This portion of the *CO1* gene was sequenced for 62 specimens from Togo, Africa. Two distinct haplotypes were found that segregated with *Spodoptera frugiperda* (Fig 3). These are identical to the rice-strain haplotype RS09 and corn-strain haplotype CS01 that are the most common forms for each strain in the United States and have both been found throughout the Western Hemisphere [35].

**Fig 4:**
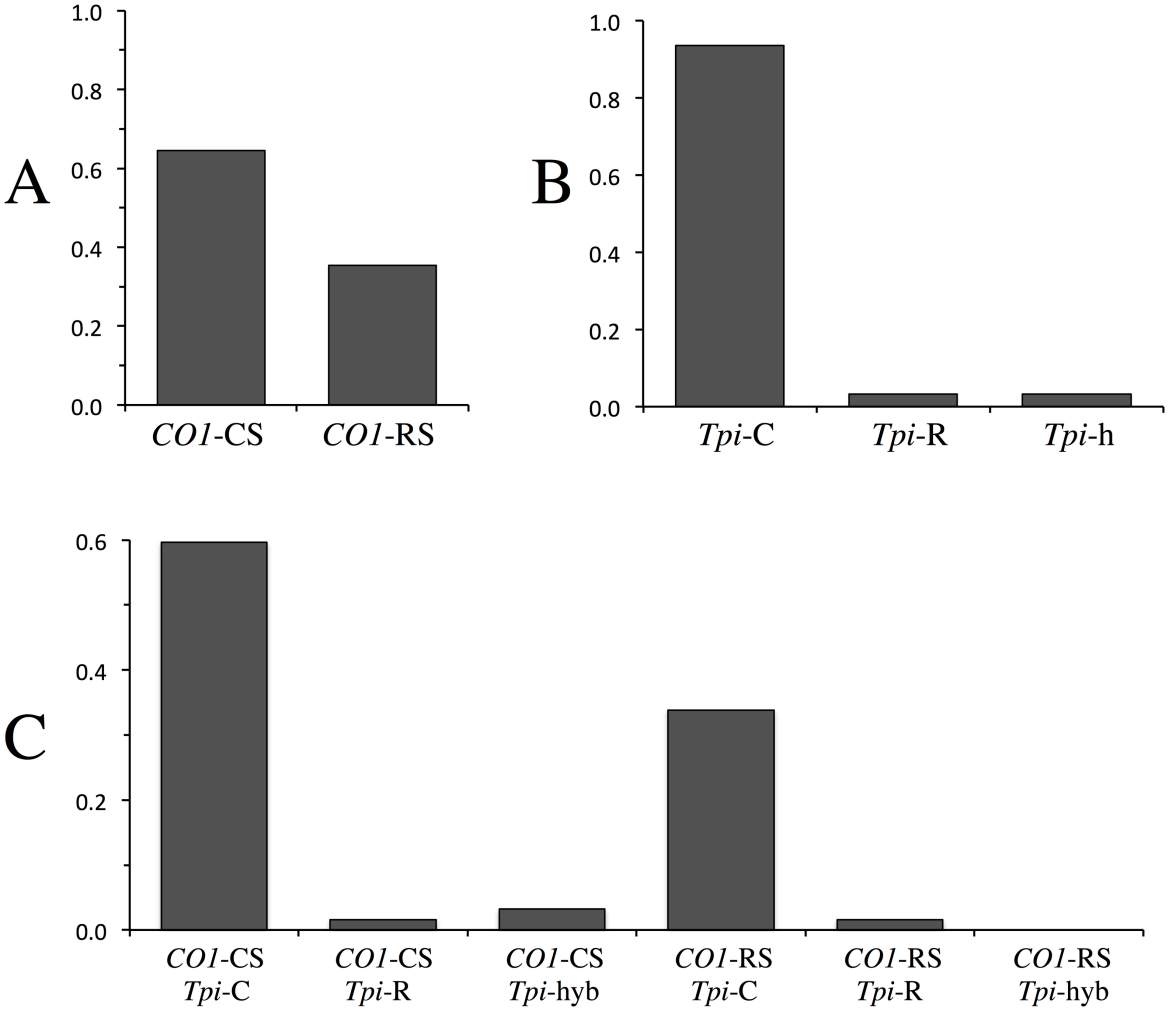
Bar graphs showing frequencies (y-axis) of different markers and haplotypes in the Togo collections. A, Frequency of *CO1* strain-specific haplotypes. B, Frequency of *Tpi* haplotypes. C, Frequency of combined *CO1* and *Tpi* haplotypes.

Of the 62 specimens analyzed, 40 were of the corn-strain (65%) and 22 of the rice-strain based on mitochondrial *CO1* haplotypes (Fig 4A). To confirm these results, we sequenced a portion of the sex-linked *Tpi* gene that previous studies suggested might be a more accurate marker of strain identity [21, 23]. Based on polymorphisms at the *Tpi* e4[165] site, 58 of the 62 specimens (94%) were corn-strain (*Tpi*-C), with two rice-strain (*Tpi*-R) and two appearing to be heterozygous for both polymorphisms (*Tpi*-h, Fig 4B).

A more stringent criterion for strain identity would be the agreement of both *CO1* and *Tpi* markers. Using this standard a majority of the Togo collection would still be designated corn-strain as 37 specimens (60%) were both *CO1*-CS and *Tpi*-C, while only one had the rice-strain concordant marker pattern of *CO1*-RS *Tpi*-R (Fig 4C). A substantial number of specimens were discordant for the two markers, with *CO1*-RS *Tpi*-C (34%) more frequently found than the reciprocal *CO1*-CS *Tpi*-R pattern (2%). The two *Tpi*-h specimens that were heterozygous for the *Tpi*-C and *Tpi*-R alleles were both *CO1*-CS.

These results illustrate how the assessment of strain proportions can vary substantially depending on the methodology used. Despite this variability, the genetic marker data consistently indicate that the corn-strain is the predominant fall armyworm subpopulation present in the Togo collections

### The Togo collection has the FL-type haplotype profile

**Fig 5:**
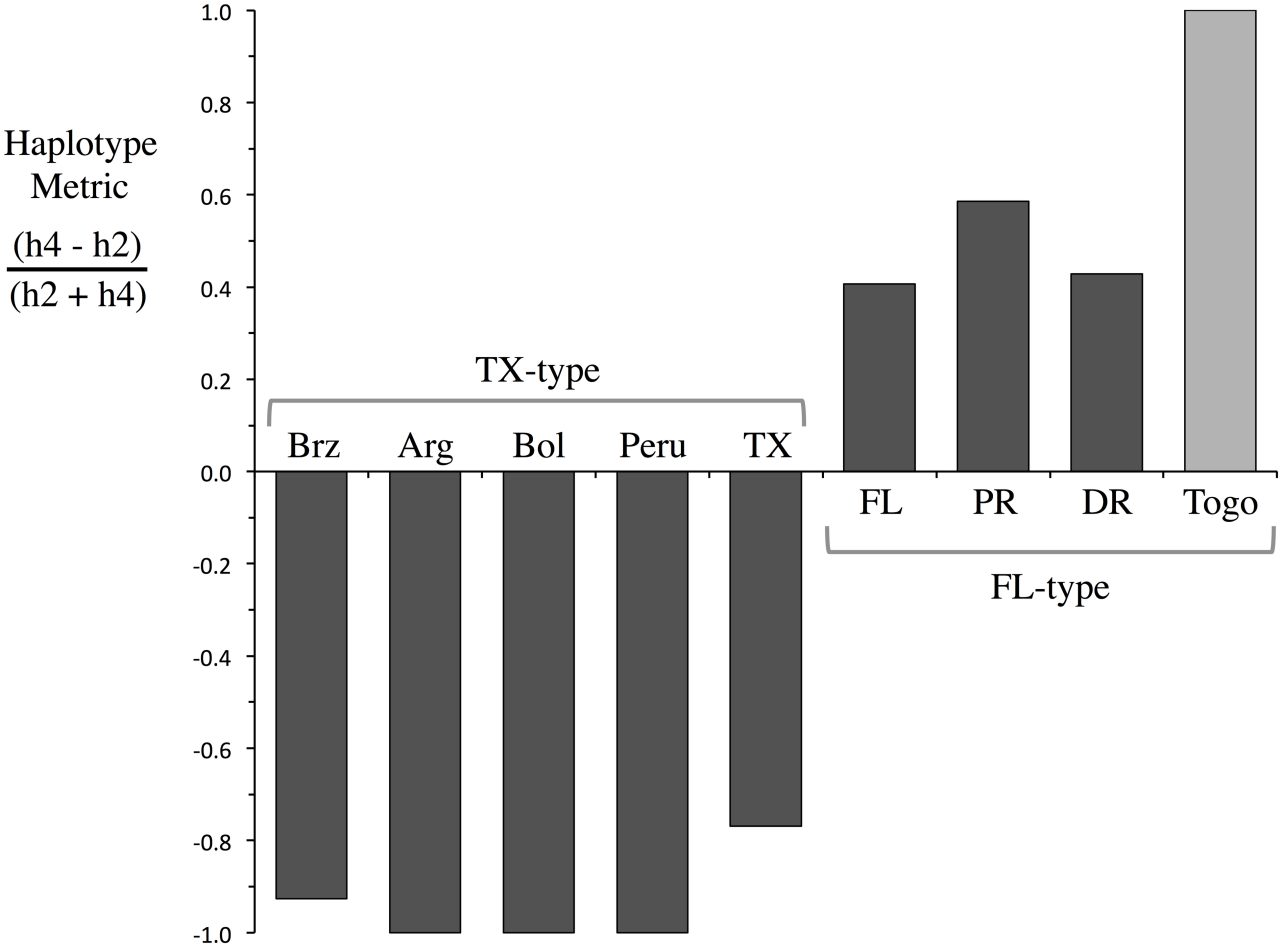
Comparison of the haplotype ratio metric from different locations in the Western Hemisphere (dark bars) and from the Togo collections (light bar). Western Hemisphere data from past studies [30, 38]. Brz, Brazil; Arg, Argentina; Bol, Bolivia; TX, Texas; FL, Florida; PR, Puerto Rico; DR, Dominican Republic.

Corn-strain *CO1* haplotypes derived from polymorphisms at sites *CO1*_1164_ and *CO1*_1287_ were shown to differ in frequency between geographically distinct subpopulations in the Western Hemisphere, designated the FL-type and TX-type [28, 29, 39]. This makes it possible to estimate which region is the most likely originating source of the Togo infestation. Five *CO1*_1164-1287_ haplotypes have been identified from Western Hemisphere fall armyworm, a single haplotype for the rice-strain and four haplotypes (h1-h4) for the corn-strain, two of which (h2 and h4) show consistent regional differences that can be quantified by a simple metric (Fig 2). The 22 Togo specimens identified as *CO1*-RS by analysis of the barcode segment all displayed the TA *CO1*_1164-1287_ haplotype, consistent with the presence of rice-strain associated mitochondria. All 40 of the *CO1*-CS specimens were of the h4 haplotype, which is the allele that predominates in the FL-type (Fig 5).

**Fig 6:**
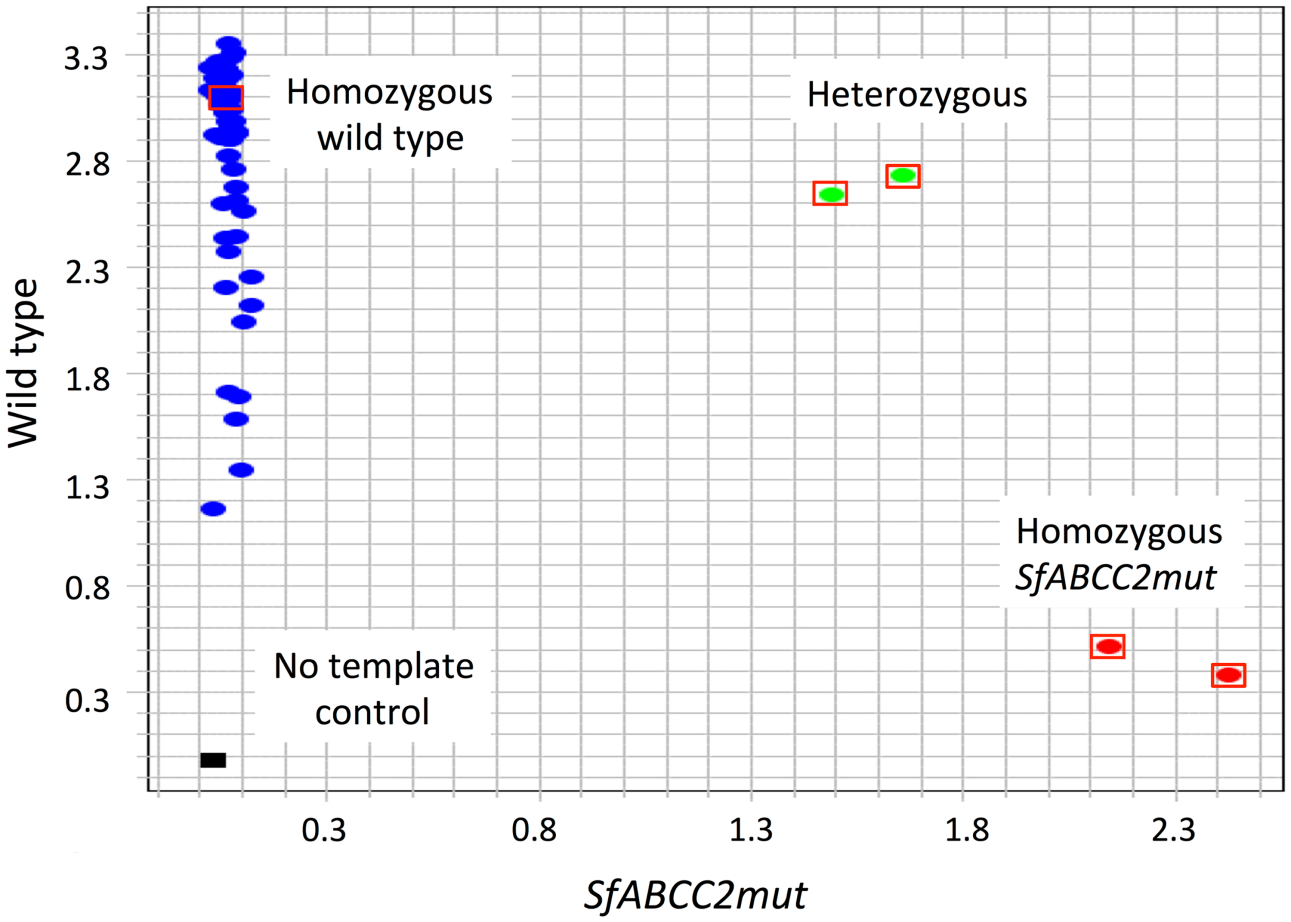
Allelic discrimination plot demonstrating the absence in the Togo collections of the *SfABCC2mut* allele associated with resistance to the Cry1Fa *Bt* toxin. Real-time PCR assays were performed using primers specific to either the *SfABCC2mut* allele or the non-mutant (wildtype) allele, with each primer tagged with a different fluorescent label. The relative levels of the *SfABCC2mut* fluorescent signal for each sample were plotted on the X-axis and the wildtype signal on the Y-axis. Homozygous *SfABCC2mut* samples (red dots) show relatively high X and low Y values. Homozygous wildtype samples (blue dots) show the converse, relatively low X and high Y values. Heterozygotes (green dots) have intermediate values. Laboratory controls for each genotype are indicated by oval with a red rectangle border. All Togo specimens (ovals with no rectangle) had low X and high Y signal. The no template control is given by the black box near the origin indicating absence of signal from either primer.

The genetic similarity of the Togo collection with Puerto Rico fall armyworm is of particular concern because of field-evolved resistance to Cry1Fa corn that arose in fall armyworm populations in Puerto Rico and was present in high frequency in Bt cornfields ([32, 40]). Genotyping for the allele linked to resistance in Puerto Rico (*SfABCC2mut*) did not detect its presence among the Togo specimens, as all individuals tested were homozygous for the wild type (susceptible) allele (Fig 6).

## Discussion

The sudden discovery of fall armyworm in Africa presents a major concern to a continent that is already periodically troubled by insufficient and unstable food supplies. The invasion of this pest presents two general problems. The first is that the introduction of a new species into an area where its normal natural enemies are not present could allow an initial period of rapid population growth and dispersion with consequent substantial impacts on agriculture. This may be the case with fall armyworm where the economic damage of infesting populations has been identified in widely dispersed regions over a short time period [5]. The second is that fall armyworm may have resistance traits new to the region that puts previously protected crops at risk. Of particular concern would be the spread of a Bt-resistance trait first detected in Puerto Rico in the first decade of this century and that may now be present in South America and North America [40–42]. As a first step to dealing with these issues we report the genetic characterization of the invading fall armyworm population in Togo to better understand what subpopulations are present, what characteristics these are likely to have, and extrapolate a possible Western Hemisphere source location.

The specimens obtained from Togo were collected from multiple regions over a six-month period in 2016. The *CO1* and *Tpi* markers both separately and in combination indicate that both strains are present in Togo, with the corn-strain predominant in the tested collection. The proportions of the two strains differed depending on the marker used, with *Tpi* giving a substantially higher corn-strain percentage than *CO1* (Fig 4). These results indicate that the Togo fall armyworm is displaying a marker pattern consistently found in the Western Hemisphere, namely that the *Tpi* strain-biased polymorphisms consistently showed a stronger correlation with plant hosts than the *CO1* haplotypes [21, 23, 26]. Overall, the data are consistent in indicating that while the rice-strain appears to be present in Africa, it is a minor component of the fall armyworm populating infesting corn in Togo. There is some evidence that the *CO1*-RS subgroup may be preferentially found in the southern portion of Togo near the coast (Fig 1). Of the 45 specimens collected at site 21 (N 06° 29.450) and more southern locations, 21 (47%) were *CO1*-RS. This compares to only 1 *CO1*-RS specimen (6%) found among the 17 collected at locations north of site 21. These numbers are small and only from a single year, so they at best describe a preliminary indication of possible differences in the geographical distribution of genetically defined subpopulations of fall armyworm. A more comprehensive and systematic survey that includes plant hosts preferred by the rice-strain is needed to determine the consistency of this observation.

**Fig 7:**
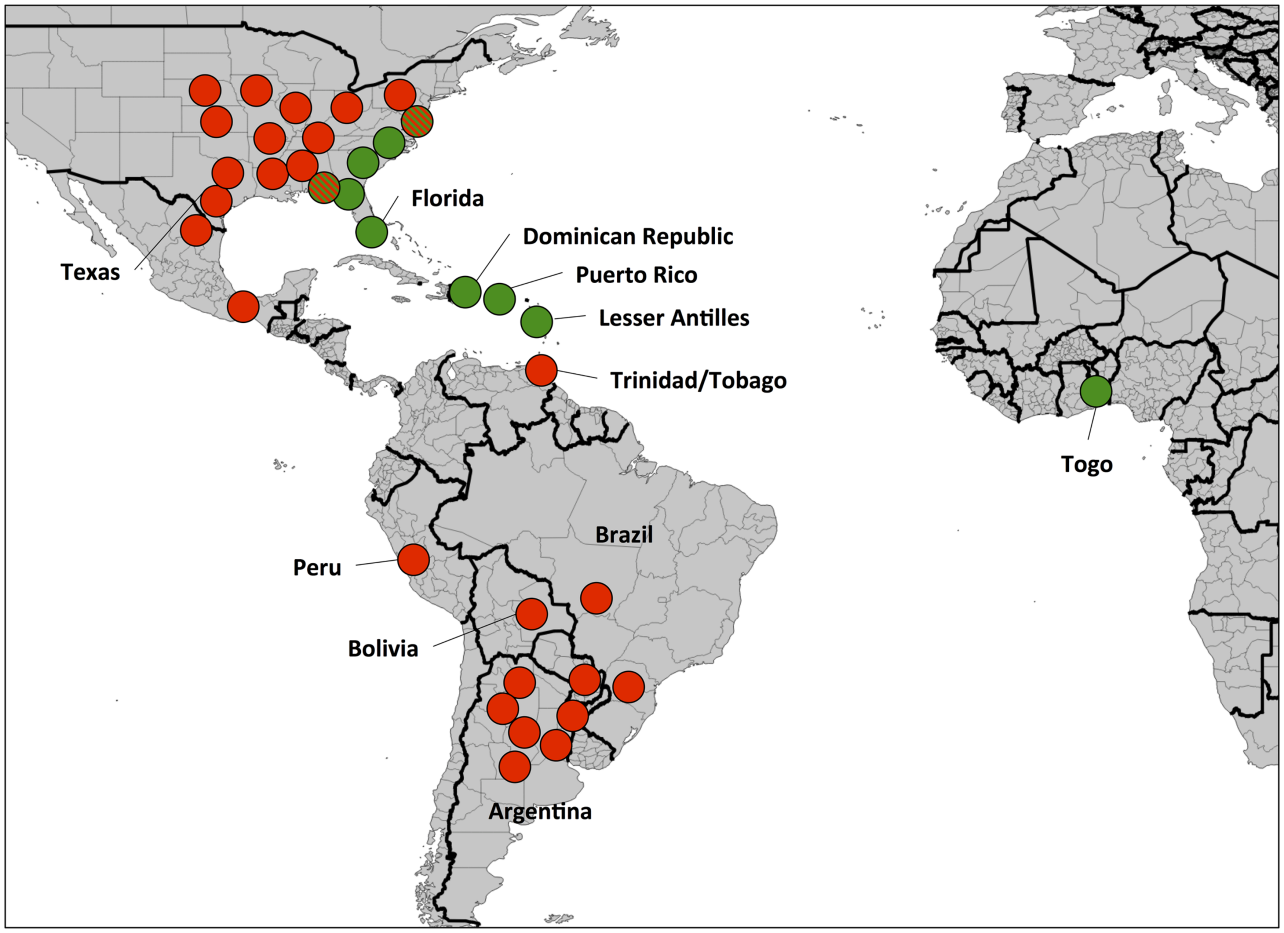
Map showing the distribution of the haplotype ratio metric in the Western Hemisphere (derived from [38]) and Togo. Circles approximate locations were fall armyworm surveys were performed with color indicating the haplotype metric found. Green circles, FL-type; red, TX-type; red and green, FAW[M] mixed profile.

The *CO1* barcode region used to identify the species and host strain of the Togo collection identified only a single rice-strain and corn-strain haplotype, both of which were the most common forms present in North American populations [35]. This low genetic variability is consistent with the results from the *CO1*_1164-1287_ analysis that identified only one of the four possible corn-strain haplotypes was present in Togo. This haplotype, h4, predominates in the FL-type subpopulation, suggesting that the Western Hemisphere source of the Togo infestation is most likely the region that extends northward from the Lesser Antilles to Puerto Rico, through Florida and includes much of the eastern coast of the United States (Fig 7). However, our finding that a marker closely linked to a Bt-resistance trait that is common in Puerto Rico populations (Banerjee et al, *in review*) was not present in Togo suggests that Puerto Rico may not be the source of the Togo infestation.

In conclusion, genetic markers provide an important resource for the investigation of fall armyworm infesting agricultural areas of Africa. Genetic analysis can confirm species identification based on morphology, is the only reliable means of identifying host strains, can provide an indication of where in the Western Hemisphere the population invading Africa might have originated, and can detect a Bt-resistance trait that could compromise the effectiveness of Bt pesticides and Bt crops as control options. The Togo population may not be representative of fall armyworm in other parts of Africa and may be susceptible to future invasive introductions, indicating the need for continued and more comprehensive genetic characterizations of African fall armyworm populations to monitor and forecast the spread of this invasive pest.
